# Supplementary material for: Urbanization, environmental stabilization and temporal persistence of bird species: a view from Latin America
Source: PeerJ. 2018 Dec 6;6:e6056. doi: 10.7717/peerj.6056 (PMC6286803; doi:10.7717/peerj.6056)
Supplement: Supplemental Information 1 — Search terms used, number of scanned papers and number of selected papers. [file peerj-06-6056-s001.pdf]

Term search	Number of papers scanned	Number of papers selected
urban* AND arthropod* AND seasonal; urban* AND insect* AND seasonal	1049	19
urban* AND arthropod* AND nocturnal; urban* AND insect* AND nocturnal	631	6
urban* AND arthropod* AND interannual; urban* AND insect* AND interannual	607	1
urban* AND artificial light	920	14
urban* AND fruit* AND interannual	301	0
urban* AND fruit* AND seasonal	415	2
urban* AND NDVI AND interannual	313	2
urban* AND NDVI AND seasonal	312	11
urban* AND land surface temperature AND interannual	315	1
urban* AND land surface temperature AND seasonal	582	24
urban* AND vegetation AND interannual	304	2
urban* AND vegetation AND seasonal	337	7
urban* AND bird* AND interannual	308	8
urban* AND bird* AND seasonal	563	4
urban* AND bird* AND nocturnal	336	18
