# Supplementary material for: Urbanization, environmental stabilization and temporal persistence of bird species: a view from Latin America
Source: PeerJ. 2018 Dec 6;6:e6056. doi: 10.7717/peerj.6056 (PMC6286803; doi:10.7717/peerj.6056)
Supplement: Supplemental Information 2 [file peerj-06-6056-s002.docx]

| Location | Main findings | Sensor | Source |
| --- | --- | --- | --- |
| Beijing, China | There was a higher seasonal change of LST in urban areas than in mixed forest areas | MODIS | Meng et al. 2009 |
| Changsha, China | The seasonal change of LST was higher in urban areas than in non-urban areas | MODIS | Zeng et al. 2010 |
| Beijing, China | There was a higher seasonal LST variation in urban areas than in forest areas | Landsat TM | Zhou et al. 2010 |
| Shanghai, China | There was a higher seasonal LST variation in urban areas than in rural areas | MODIS | Jin et al. 2011 |
| Shanghai, China | There was a higher seasonal LST variation in urban areas than in forest and shrubland areas | Landsat TM and ETM+ | Li et al. 2012 |
| Jinan, China | There was a higher seasonal LST variation in urban areas than in forest and shrubland areas | Landsat TM and ETM+ | Meng and Liu 2013 |
| Beijing, China | Urban areas had more seasonal change of LST than forest areas | MODIS | Qiao et al. 2013 |
| Yangtze River, China | Similar seasonal variation of LST between urban and agricultural areas | MODIS | Du et al. 2016 |
| Beijing, China | Similar seasonal variation of LST between urban areas and forest areas | Landsat TM | Fu and Weng 2016a |
| Changchun, China | The smallest green areas showed the highest seasonal change of LST | Landsat OLI/TIRS | Yang et al. 2017 |
| Beijing, China | There was a higher seasonal change of LST in urban areas than in cropland areas | MODIS | Zhao et al. 2017 |
| Delhi, India | There was a higher seasonal change of LST in urban areas than in vegetated areas | Landsat TM | Singh et al. 2014 |
| Delhi, India | There was a higher seasonal change of LST in agricultural areas than in urban areas | Landsat TM and MODIS | Chakraborty et al. 2015 |
| Abu Dhabi, UAE | Downtown areas had lower seasonal change of LST than sand dunes areas, but higher change of NDVI | MODIS/ASTER | Lazzarini et al. 2013 |
| Tehran, Iran | There was a higher seasonal change of LST in urban areas than in agriculture areas | MODIS | Haashemi et al. 2016 |
| Baghdad, Iraq | There was a higher seasonal change of LST in urban areas than in periurban areas | Landsat TM | Naem et al. 2016 |
| English Bazar, Egypt | There was a lower seasonal change of LST in urban areas than in fallow land areas | Landsat TM and OLI/TIRS | Pal and Ziaul 2017 |
| Lagos, Nigeria | During the day, there was a higher seasonal change of LST in urban areas than in forest areas | MODIS | Ayandale 2017 |
| Milan, Bologna, Florence, Rome; Italy | There was a higher seasonal change of LST in urban areas than in non-urban areas | MODIS | Morabito et al. 2016 |
| Los Angeles, USA | Urban areas showed the lowest yearly amplitude of LST | Landsat TM | Weng and Fu 2014 |
| Atlanta, USA | There was a higher seasonal change of LST after conversion of evergreen forest to urban areas | Landsat TM and ETM+ | Fu and Weng 2016b |
| Columbus, USA | Green areas reduced summer temperature and increased winter temperatures | Landsat TM | Chun and Guldmann 2018 |
| Phoenix, USA | There was a higher seasonal change of LST in desert areas than in commercial areas | 0,5 m above ground | Stabler et al. 2005 |
| Monte Hermoso, Argentina | There was a lower seasonal change of LST in the urban area than in the periphery of the city | Landsat TM and ETM+ | Ferrelli et al. 2018 |

Abbreviations: LST, Land surface temperature; NDVI, Normalized Difference vegetation index; MODIS, Moderate resolution imaging spectroradiometer; ASTER, Advanced Spaceborne Thermal Emission and Reflection Radiometer; TM, Thematic Mapper; ETM+, Enhanced Thematic Mapper plus; OLI/TIRS, Operational Land Imager/Thermal Infrared Sensor

Spatial resolution: MODIS and ASTER (1000 m), Landsat TM (120 m), Landsat ETM+ (60 m), Landsat OLI/TIRS (100 m).

References

Ayanlade, A. (2017). Variations in urban surface temperature: an assessment of land use change impacts over Lagos metropolis. *Weather*, *72*(10), 315-319.

Chakraborty, S. D., Kant, Y., & Mitra, D. (2015). Assessment of land surface temperature and heat fluxes over Delhi using remote sensing data. *Journal of environmental management*, *148*, 143-152.

Chun, B., & Guldmann, J. M. (2018). Impact of greening on the urban heat island: Seasonal variations and mitigation strategies. *Computers, Environment and Urban Systems*.

Du, H., Wang, D., Wang, Y., Zhao, X., Qin, F., Jiang, H., & Cai, Y. (2016). Influences of land cover types, meteorological conditions, anthropogenic heat and urban area on surface urban heat island in the Yangtze River Delta Urban Agglomeration. *Science of the Total Environment*, *571*, 461-470.

Ferrelli, F., Cisneros, M. A. H., Delgado, A. L., & Piccolo, M. C. (2018). Spatial and temporal analysis of the LST-NDVI relationship for the study of land cover changes and their contribution to urban planning in Monte Hermoso, Argentina. *Documents d'anàlisi geogràfica*, *64*(1), 25-47.

Fu, P., & Weng, Q. (2016a). Consistent land surface temperature data generation from irregularly spaced Landsat imagery. *Remote Sensing of Environment*, *184*, 175-187.

Fu, P., & Weng, Q. (2016b). A time series analysis of urbanization induced land use and land cover change and its impact on land surface temperature with Landsat imagery. *Remote Sensing of Environment*, *175*, 205-214.

Haashemi, S., Weng, Q., Darvishi, A., & Alavipanah, S. K. (2016). Seasonal variations of the surface urban heat island in a semi-arid city. *Remote Sensing, 8*(4), 352.

Jin, M. S., Kessomkiat, W., & Pereira, G. (2011). Satellite-observed urbanization characters in Shanghai, China: aerosols, urban heat island effect, and land–atmosphere interactions. *Remote Sensing*, *3*(1), 83-99.

Lazzarini, M., Marpu, P. R., & Ghedira, H. (2013). Temperature-land cover interactions: The inversion of urban heat island phenomenon in desert city areas. *Remote Sensing of Environment*, *130*, 136-152.

Li, Y. Y., Zhang, H., & Kainz, W. (2012). Monitoring patterns of urban heat islands of the fast-growing Shanghai metropolis, China: Using time-series of Landsat TM/ETM+ data. *International Journal of Applied Earth Observation and Geoinformation*, *19*, 127-138.

Meng, D., Gong, H., Li, X., Zhao, W., Gong, Z., Zhu, L., & Hu, D. (2009, July). Study of thermal environment based on remote sensing in Beijing-capital zone. In *Geoscience and Remote Sensing Symposium, 2009 IEEE International, IGARSS 2009*(Vol. 3, pp. III-353). IEEE.

Meng, F., & Liu, M. (2013). Remote-sensing image-based analysis of the patterns of urban heat islands in rapidly urbanizing Jinan, China. *International journal of remote sensing*, *34*(24), 8838-8853.

Morabito, M., Crisci, A., Messeri, A., Orlandini, S., Raschi, A., Maracchi, G., & Munafò, M. (2016). The impact of built-up surfaces on land surface temperatures in Italian urban areas. *Science of the Total Environment*, *551*, 317-326.

Naem, M., Corner, R., & Dewan, A. (2016). Diurnal and Seasonal Surface Temperature Variations: A Case Study in Baghdad. In *CEUR Workshop Proceedings* (Vol. 1570, pp. 65-70).

Pal, S., & Ziaul, S. (2017). Detection of land use and land cover change and land surface temperature in English Bazar urban centre. *The Egyptian Journal of Remote Sensing and Space Science*, *20*(1), 125-145.

Qiao, Z., Tian, G., & Xiao, L. (2013). Diurnal and seasonal impacts of urbanization on the urban thermal environment: a case study of Beijing using MODIS data. *ISPRS Journal of Photogrammetry and Remote Sensing*, *85*, 93-101.

Singh, R. B., Grover, A., & Zhan, J. (2014). Inter-seasonal variations of surface temperature in the urbanized environment of Delhi using Landsat thermal data. *Energies*, *7*(3), 1811-1828.

Stabler, L. B., Martin, C. A., & Brazel, A. J. (2005). Microclimates in a desert city were related to land use and vegetation index. *Urban Forestry & Urban Greening*, *3*(3-4), 137-147.

Weng, Q., & Fu, P. (2014). Modeling annual parameters of clear-sky land surface temperature variations and evaluating the impact of cloud cover using time series of Landsat TIR data. *Remote Sensing of Environment*, *140*, 267-278.

Yang, C., He, X., Wang, R., Yan, F., Yu, L., Bu, K., Yang, J., Chang, L., & Zhang, S. (2017). The effect of urban green spaces on the urban thermal environment and its seasonal variations. *Forests*, *8*(5), 153.

Zeng, Y., Huang, W., Zhan, F., Zhang, H., & Liu, H. (2010). Study on the urban heat island effects and its relationship with surface biophysical characteristics using MODIS imageries. *Geo-spatial Information Science*, *13*(1), 1-7.

Zhao, G., Dong, J., Liu, J., Zhai, J., Cui, Y., He, T., & Xiao, X. (2017). Different patterns in daytime and nighttime thermal effects of urbanization in Beijing-Tianjin-Hebei urban agglomeration. *Remote Sensing*, *9*(2), 121.

Zhou, J., Hu, D., & Weng, Q. (2010). Analysis of surface radiation budget during the summer and winter in the metropolitan area of Beijing, China. *Journal of Applied Remote Sensing*, *4*(1), 0435
