# Supplementary material for: Urbanization, environmental stabilization and temporal persistence of bird species: a view from Latin America
Source: PeerJ. 2018 Dec 6;6:e6056. doi: 10.7717/peerj.6056 (PMC6286803; doi:10.7717/peerj.6056)
Supplement: Supplemental Information 3 [file peerj-06-6056-s003.docx]

| Location | Main findings | Sensor | Source |
| --- | --- | --- | --- |
| Asia, Europe, and North America | Longer growing season in urban than in natural areas | MODIS | Zhang et al. 2004 |
| Taiwan | The length of the growing season was shorter in urban than in forest | MODIS | Chang et al. 2011 |
| Pearl River Delta, China | Least seasonal change of net primary productivity in urban areas | Landsat TM and ETM+; and MODIS | Yan et al. 2018 |
| China | The length of the growing season was higher in urban than in rural areas, and it increased with the UHI effect | MODIS | Zhou et al. 2016 |
| Shanghai, China | Longer length of growing season in urban than in rural areas | Landsat | Qiu et al. 2017 |
| Uttarakhand region, India | There was a higher seasonal change of NDVI in urban than in evergreen forest during 2009-2014 period | MODIS | Mishra and Chaudhuri 2015 |
| USA | Several cities showed an extended length of the growing season | AVHRR | Imhoff et al. 2000 |
| Eastern USA | Urban areas extended their growing season 7.6 days in comparison to deciduous broadleaf forests | AVHRR | White et al. 2002 |
| USA | Urban areas had a lower seasonal variation of primary productivity than grassland and shrubland areas | MODIS | Coops et al. 2009 |
| San Fernando Valley, USA | Irrigated urban areas had a constant NDVI through the year in comparison to non-irrigated areas | Landsat TM | Johnson and Belitz 2012 |
| Phoenix, USA | Urban vegetation stayed photosynthetically active for longer periods than native vegetation | MODIS | Buyantuyev and Wu 2012 |
| Brentwood, USA | Primary productivity fluctuated less in urban than in natural and agricultural areas | MODIS | Leong and Roderick 2015 |
| King, Polk, and Baltimore county; USA | Urban areas showed an extended length of the growing season in comparison to rural areas | Landsat TM, ETM+ , and OLI/TIRS; MODIS | Li et al. 2017 |
| Phalaborwa, South Africa | There was a lower seasonal change of NDVI in urban than in natural areas | MODIS | Coetzee and Chown 2016 |
| Mar del Plata, Argentina | There was a lower seasonal change of NDVI in urban than in rural areas | MODIS | Leveau et al. 2018 |

Abbreviations: UHI, Urban heat island; NDVI, Normalized Difference vegetation index; MODIS, Moderate resolution imaging spectroradiometer; AVHRR, Advanced Very High Resolution Radiometer; TM, Thematic Mapper; ETM+, Enhanced Thematic Mapper plus; OLI/TIRS, Operational Land Imager/Thermal Infrared Sensor.

Spatial resolution: AVHRR (1000 m); MODIS (250 m); Landsat sensors (30 m).

References

Buyantuyev, A., & Wu, J. (2012). Urbanization diversifies land surface phenology in arid environments: interactions among vegetation, climatic variation, and land use pattern in the Phoenix metropolitan region, USA. *Landscape and Urban Planning*, *105*(1), 149-159.

Chang, C. T., Lin, T. C., Wang, S. F., & Vadeboncoeur, M. A. (2011). Assessing growing season beginning and end dates and their relation to climate in Taiwan using satellite data. *International Journal of Remote Sensing*, *32*(18), 5035-5058.

Coetzee, B. W., & Chown, S. L. (2016). Land‐use change promotes avian diversity at the expense of species with unique traits. *Ecology and Evolution*, *6*(21), 7610-7622.

Coops, N. C., Wulder, M. A., & Iwanicka, D. (2009). Exploring the relative importance of satellite-derived descriptors of production, topography and land cover for predicting breeding bird species richness over Ontario, Canada. *Remote Sensing of Environment*, *113*(3), 668-679.

Imhoff, M. L., Tucker, C. J., Lawrence, W. T., & Stutzer, D. C. (2000). The use of multisource satellite and geospatial data to study the effect of urbanization on primary productivity in the United States. *IEEE Transactions on Geoscience and Remote Sensing*, *38*(6), 2549-2556.

Johnson, T. D., & Belitz, K. (2012). A remote sensing approach for estimating the location and rate of urban irrigation in semi-arid climates. *Journal of Hydrology*, *414*, 86-98.

Leong, M., & Roderick, G. K. (2015). Remote sensing captures varying temporal patterns of vegetation between human-altered and natural landscapes. *PeerJ*, *3*, e1141.

Leveau, L. M., Isla, F. I., & Bellocq, M. I. (2018). Predicting the seasonal dynamics of bird communities along an urban-rural gradient using NDVI. *Landscape and Urban Planning*, *177*, 103-113.

Li, X., Zhou, Y., Asrar, G. R., & Meng, L. (2017). Characterizing spatiotemporal dynamics in phenology of urban ecosystems based on Landsat data. *Science of the Total Environment*, *605*, 721-734.

Mishra, N. B., & Chaudhuri, G. (2015). Spatio-temporal analysis of trends in seasonal vegetation productivity across Uttarakhand, Indian Himalayas, 2000–2014. *Applied Geography*, *56*, 29-41.

Qiu, T., Song, C., & Li, J. (2017). Impacts of Urbanization on Vegetation Phenology over the Past Three Decades in Shanghai, China. *Remote Sensing*, *9*(9), 970.

White, M. A., Nemani, R. R., Thornton, P. E., & Running, S. W. (2002). Satellite evidence of phenological differences between urbanized and rural areas of the eastern United States deciduous broadleaf forest. *Ecosystems*, *5*(3), 260-273.

Yan, Y., Liu, X., Wang, F., Li, X., Ou, J., Wen, Y., & Liang, X. (2018). Assessing the impacts of urban sprawl on net primary productivity using fusion of Landsat and MODIS data. *Science of The Total Environment*, *613*, 1417-1429.

Zhang, X., Friedl, M. A., Schaaf, C. B., & Strahler, A. H. (2004). Climate controls on vegetation phenological patterns in northern mid‐and high latitudes inferred from MODIS data. *Global change biology*, *10*(7), 1133-1145.

Zhou, D., Zhao, S., Zhang, L., & Liu, S. (2016). Remotely sensed assessment of urbanization effects on vegetation phenology in China's 32 major cities. *Remote Sensing of Environment*, *176*, 272-281.
