# Supplementary material for: Urbanization, environmental stabilization and temporal persistence of bird species: a view from Latin America
Source: PeerJ. 2018 Dec 6;6:e6056. doi: 10.7717/peerj.6056 (PMC6286803; doi:10.7717/peerj.6056)
Supplement: Supplemental Information 4 [file peerj-06-6056-s004.docx]

| Location | Main results | Periods compared | Source |
| --- | --- | --- | --- |
| Burzaco, Argentina | Sarcophagid Diptera abundance did not show significant changes through the year in suburban habitats compared with urban and rural habitats | months | Mulieri et al. 2011 |
| Phoenix, USA | Arthropod abundance did not show a significant response to temperature in industrial and agriculture areas | months | McIntyre et al. 2001 |
| Phoenix, USA | Aphid abundance had a lower seasonal change in irrigated residential areas than in natural and non-irrigated areas | seasons | Andrade et al. 2017 |
| Sorø, Denmark | The activity of carabid species started earlier in urban habitats than in rural forests | weeks | Lövei et al. 2018 |
| Switzerland | The annual variation of hoverfly abundance was lower in urban areas than in rural areas | months | Luder et al. 2018 |
| Bulgary | Caterpillar abundance had a lower seasonal change in urban areas compared to forest areas | months | Seress et al. 2018 |
| Kolkata, India | *Aedes aegypti* larval frequency was more stable in urban habitats than in rural habitats, whereas *A. albopictus* larval frequency was more stable in rural habitats | months | Banerjee et al. 2015 |
| Penang island, Malaysia | There was a lower seasonal variation of *A. aegypti* and *A. albopictus* positive containers occupied by larvae in urban areas than in rural areas | months | Saifur et al. 2012 |
| Latin America | Outdoor containers filled with water during the dry season were important for the production of pupae of *A. aegypti* | wet-dry seasons | Quintero et al. 2014 |
| Buenos Aires, Argentina | Broken water pipes allowed permanent flooded pools where mosquitoes presence was more stable along the year than in natural filled by rain pools | months | Quiroga et al. 2013 |
| Phoenix, USA | Monthly variation of arthropod biomass in Phoenix was similar between urban and desert habitats | months | Davies et al. 2016 |
| Buenos Aires, Argentina | Adult oviposition of *A. aegypti* was longer along the year in areas of houses than in areas of high buildings in Buenos Aires city | months | Carbajo et al. 2004 |
| North-western Italy | Proximity to urban areas did not affect the phenology of *Culex pipiens* | weeks | Rosá et al. 2014 |
| Chiang Mai, Thailand | Seasonal variation in egg numbers of *A. aegyipti* and *A. albopictus* was similar between urban and rural areas | months | Mogi et al. 1988 |
| Eastern USA | The annual flight activity of the Dogwood borer (*Synanthedon scitula*) was similar between urban areas and apple orchards | weeks | Bergh et al. 2009 |
| Sisaket, Thailand | Seasonal variation of positive ovitraps and containers infested with larvae of *A. aegypti* was higher in urban areas than in industrial areas | months | Baruah and Dutta 2013 |
| Dibrugarh, India | Seasonal variation of positive ovitraps and containers infested with larvae of *A. aegypti* and *A. albopictus* was higher in urban areas than in rural areas | winter, summer, and rainy | Wongkoon et al. 2013 |
| Canton of Aargau, Switzerland | Butterfly appearance in urban areas was delayed in comparison to non-urban areas | months | Altermatt, 2012 |
| Ohio, USA | Urbanization and climate change delayed the butterfly appearance | months | Diamond et al., 2014 |
| Natal, Brazil | The seasonal variation of butterfly abundance in urban parks was related to annual fluctuations in precipitation and humidity, but in a nearby natural reserve it was unrelated with that factors | months | Oliveira et al. 2018 |

References

Altermatt, F. (2012). Temperature‐related shifts in butterfly phenology depend on the habitat. *Global Change Biology*, *18*(8), 2429-2438.

Andrade, R., Bateman, H. L., & Kang, Y. (2017). Seasonality and land cover characteristics drive aphid dynamics in an arid city. *Journal of Arid Environments*, *144*, 12-20.

Banerjee, S., Aditya, G., & Saha, G. K. (2015). Household wastes as larval habitats of dengue vectors: comparison between urban and rural areas of Kolkata, India. *PloS one*, *10*(10), e0138082.

Baruah, S., & Dutta, P. (2013). Seasonal prevalence of Aedes aegypti in urban and industrial areas of Dibrugarh district, Assam. *Trop Biomed*, *30*(434), 43.

Bergh, J. C., Leskey, T. C., Walgenbach, J. F., Klingeman, W. E., Kain, D. P., & Zhang, A. (2009). Dogwood borer (Lepidoptera: Sesiidae) abundance and seasonal flight activity in apple orchards, urban landscapes, and woodlands in five eastern states. *Environmental entomology*, *38*(3), 530-538.

Carbajo, A. E., Gomez, S. M., Curto, S. I., & Schweigmann, N. J. (2004). Variación espacio-temporal del riesgo de transmisión de dengue en la Ciudad de Buenos Aires. *Medicina (Buenos Aires)*, *64*(3), 231-234.

Davies, S., Lane, S., Meddle, S. L., Tsutsui, K., & Deviche, P. (2016). The ecological and physiological bases of variation in the phenology of gonad growth in an urban and desert songbird. *General and comparative endocrinology*, *230*, 17-25.

Diamond, S. E., Cayton, H., Wepprich, T., Jenkins, C. N., Dunn, R. R., Haddad, N. M., & Ries, L. (2014). Unexpected phenological responses of butterflies to the interaction of urbanization and geographic temperature. *Ecology*, *95*(9), 2613-2621.

Lövei, G. L., Elek, Z., Howe, A., & Engaard, M. (2018). The use of percentile-percentile plots to compare differences in seasonal dynamics, illustrated by the case of ground beetles (Coleoptera, Carabidae) reacting to urbanisation. *Community Ecology*, *19*(1), 1-8.

Luder, K., Knop, E., & Menz, M. H. (2018). Contrasting responses in community structure and phenology of migratory and non‐migratory pollinators to urbanization. *Diversity and Distributions*.

McIntyre, N. E., Rango, J., Fagan, W. F., & Faeth, S. H. (2001). Ground arthropod community structure in a heterogeneous urban environment. *Landscape and urban planning*, *52*(4), 257-274.

Mogi, M., Khamboonruang, C., Choochote, W., & Suwanpanit, P. (1988). Ovitrap surveys of dengue vector mosquitoes in Chiang Mai, northern Thailand: seasonal shifts in relative abundance of Aedes albopictus and Ae. aegypti. *Medical and Veterinary Entomology*, *2*(4), 319-324.

Mulieri, P. R., Patitucci, L. D., Schnack, J. A., & Mariluis, J. C. (2011). Diversity and seasonal dynamics of an assemblage of sarcophagid Diptera in a gradient of urbanization. *Journal of Insect Science*, *11*(1).

Oliveira, I. F., Lion, M. B., & Cardoso, M. Z. (2018). A plaza too far: High contrast in butterfly biodiversity patterns between plazas and an urban reserve in Brazil. *Landscape and Urban Planning*, *180*, 207-216.

Quintero, J., Brochero, H., Manrique-Saide, P., Barrera-Pérez, M., Basso, C., Romero, S., Caprara, A., De Lima Cunha, J. C., Beltrán – Ayala, E., Mitchell-Foster, K., Kroeger, A., Sommerfeld, J., & Kroeger, A. (2014). Ecological, biological and social dimensions of dengue vector breeding in five urban settings of Latin America: a multi-country study. *BMC infectious diseases*, *14*(1), 38.

Quiroga, L., Fischer, S., & Schweigmann, N. (2013). Immature mosquitoes associated with urban parklands: Implications for water and mosquito management. *Journal of the American Mosquito Control Association, 29*(1), 27-32.

Rosà, R., Marini, G., Bolzoni, L., Neteler, M., Metz, M., Delucchi, L., Chadwick, E. A., Balbo, L., Mosca, A., Giacobini, M., Bertolotti, L., & Bertolotti, L. (2014). Early warning of West Nile virus mosquito vector: climate and land use models successfully explain phenology and abundance of Culex pipiens mosquitoes in north-western Italy. *Parasites & vectors*, *7*(1), 269.

Saifur, R. G., Hassan, A. A., Dieng, H., Ahmad, H., Salmah, M. R. C., Satho, T., Saad, A. R., & Vargas, R. E. M. (2012). Update on temporal and spatial abundance of dengue vectors in Penang, Malaysia. *Journal of the American Mosquito Control Association*, *28*(2), 84-92.

Seress, G., Hammer, T., Bókony, V., Vincze, E., Preiszner, B., Pipoly, I., Sinkovics, C., Evans, K. L., & Liker, A. (2018). Impact of urbanization on abundance and phenology of caterpillars and consequences for breeding in an insectivorous bird. *Ecological Applications*.

Wongkoon, S., Jaroensutasinee, M., & Jaroensutasinee, K. (2013). Distribution, seasonal variation & dengue transmission prediction in Sisaket, Thailand. *The Indian journal of medical research*, *138*(3), 347.
